# Supplementary material for: The Relationship Between Dietary Patterns, Cognition, and Cardiometabolic Health in Healthy, Older Adults
Source: Nutrients. 2024 Nov 14;16(22):3890. doi: 10.3390/nu16223890 (PMC11597354; doi:10.3390/nu16223890)
Supplement: Supplementary file 1 [file nutrients-16-03890-s001.zip › nutrients-3311736-supplementary.pdf]

## Supplementary Material

**Table 1**

Test-retest reliability coefficients for CANTAB neuropsychological assessments

| Test Name | Metric | Test-Retest Reliability Coefficient (r) |
|---|---|---|
| Paired Associates Learning (PAL) | Stages Completed | 0.87* |
| | Total Errors | 0.68 |
| Reaction Time (RTI) | Simple Reaction Time | 0.54 |
| | Choice Reaction Time | 0.57 |
| | Movement Time | 0.73 |
| Multitasking Test (MTT) | Not Listed | Not Available |
| Verbal Recognition Memory (VRM) | Immediate and Delayed Recall | Not Available |
| One Touch Stockings of Cambridge (OTS) | Initial Thinking Time | 0.69 |
| | Subsequent Thinking Time | 0.64* |
| | Minimum Moves | 0.64 |

*Note*. The table presents test-retest reliability coefficients for selected tasks from the Cambridge Neuropsychological Test Automated Battery (CANTAB; Cambridge Cognition, 2008). The asterisks (*) indicate the use of Spearman's rho, a non-parametric measure of statistical dependence between two variables.

**Table 2**

Grouping of food items for PCA

| **Whole grains** | **Refined grains** | **Sweet snacks** | **Savoury snacks** | **Discretionary dairy** |
|---|---|---|---|---|
| Cooked porridge<br>Brown bread | English muffin<br>Savoury biscuits<br>White bread<br>Other bread<br>Muesli<br>Pasta<br>Noodles<br>Rice<br>Other grains<br>e.g., couscous<br>Cereal | Cakes<br>Puddings<br>Sweet biscuits<br>Choc biscuits<br>Fruit bars<br>Snack bars<br>Muesli bars<br>Sweet combination<br>Snacks<br>Ice blocks<br>Lollies<br>Jam & honey<br>Nut spread | Savoury<br>combination snacks<br>Snack noodles<br>Chips<br>Potato chips<br>Savoury pastries | Flavoured milk<br>Cream or sour cream<br>Ice cream<br>Frozen yoghurt<br>Ice block (creamy)<br>Cheese spread |
| **Dairy (normal)** | **Seafood** | **Chicken with veg** | **Chicken without veg** | **Meat with veg** |
| Cottage cheese<br>Cheese<br>Yoghurt<br>Full fat milk<br>Low fat milk<br>Skim milk | Fresh fish<br>Canned tuna<br>Other seafood | Chicken pieces<br>Plain chicken | Chicken pieces<br>Plain chicken | Beef / lamb<br>Plain meat<br>Pork pieces<br>Plain pork |
| **Meat without veg** | **Processed meat** | **Fried protein** | **Potato** | **Red-Yellow vegetables** |
| Mince dish<br>Beef / lamb<br>Plain meat<br>Pork pieces<br>Plain pork | Bacon / ham<br>Sausage<br>Salami / Devon | Chicken<br>Fish | Potato<br>Hot chips cooked at home | Carrots<br>Pumpkin<br>Sweet potato<br>Tomatoes |
| **Other vegetables** | **Green leafy vegetables** | **Cruciferous vegetables** | **Legumes** | **Fresh fruit** |
| Zucchini<br>Capsicum<br>Mushrooms<br>Celery<br>Onion<br>Corn | Spinach<br>Lettuce | Broccoli<br>Cauliflower<br>Cabbage | Green beans<br>Peas<br>Soybeans<br>Baked beans<br>Other beans | Fruit salad<br>Apple<br>Pear<br>Banana<br>Melon<br>Pineapple<br>Peach<br>Mango<br>Orange |
| **Alcohol** | **Sugary beverages** | **Fast fried food** | **Condiments** | **Other oils** |
| Beer<br>Wine<br>Spirits | Soft drink<br>Fruit juice<br>Cordial | Hamburger<br>Hot dog<br>Pizza<br>Pie / Sausage roll<br>Hot chips<br>Hash brown<br>Tacos | Salad dressing<br>Low fat salad dressing<br>Vegemite<br>Tomato sauce | Vegetable<br>Canola<br>Sunflower<br>Peanut<br>Coconut |
| **Eggs** | **Diet soft drink** | **Margarine** | **Avocado** | **Tea / Coffee** |
| **Canned fruit** | **Dried fruit** | **Butter/lard** | **Chocolate** | **Nuts** |
| **Other milk**<br>(rice milk, soy milk) | **Olive oil** | **Soup**<br>(creamy / not creamy) | **Water** | **Liver**<br>(e.g., beef / chicken) |

**Table 3**

Variance Inflation Factors (VIF) for dietary, demographic, and physical activity variables across cognitive outcomes

| **Variable** | **Long-term memory** | **Short-term memory** | **Executive function** | **Processing speed** |
|---|---|---|---|---|
| Age | 1.03 | 1.04 | 1.03 | 1.03 |
| Sex | 1.79 | 1.94 | 1.84 | 1.85 |
| Education | 1.04 | 1.05 | 1.04 | 1.04 |
| MVPA | 1.14 | 1.19 | 1.15 | 1.13 |
| Energy intake | 3.95 | 4.32 | 3.97 | 4.01 |
| Meat- dominant diet | 1.75 | 1.78 | 1.75 | 1.75 |
| Plant-dominant diet | 2.17 | 2.31 | 2.17 | 2.16 |

*Note.* MVPA = Moderate to Vigorous Physical Activity; Higher VIF values suggest moderate multicollinearity.

**Table 4**

Variance Inflation Factors (VIF) for moderation analyses

| **Variable** | **Long-term memory** | **Short-term memory** | **Executive function** | **Processing speed** |
|---|---|---|---|---|
| Age | 1.06 | 1.09 | 1.07 | 1.07 |
| Sex | 1.86 | 2.00 | 1.91 | 1.91 |
| Education | 1.06 | 1.07 | 1.06 | 1.06 |
| MVPA | 1.21 | 1.23 | 1.22 | 1.21 |
| Energy intake | 4.13 | 4.56 | 4.18 | 4.20 |
| Meat-dominant diet | 4.03 | 4.31 | 3.94 | 4.00 |
| Plant-dominant diet | 3.24 | 3.61 | 3.27 | 3.27 |
| Western-dominant diet | 4.45 | 5.08 | 4.40 | 4.42 |
| MetSSS | 1.21 | 1.22 | 1.21 | 1.21 |
| Meat**MetSSS | 4.22 | 4.65 | 3.98 | 4.19 |
| Plant**MetSSS | 2.44 | 2.46 | 2.45 | 2.45 |
| Western**MetSSS | 4.15 | 4.84 | 4.02 | 4.14 |

**Table 5**

Sensitivity analysis using the raw data for regression outcomes for cognitive functions across different dietary patterns and three progressive model adjustments. Model 1 includes only dietary patterns; Model 2 adjusts for age, sex, and education; and Model 3 also adjusts for energy intake and physical activity.

| | | | Plant-dominant Diet | Meat-dominant Diet | Western-style Diet | Age | Sex | Education | Energy intake | MVPA |
|---|---|---|---|---|---|---|---|---|---|---|
| Long term Memory | Model 1 | β | 0.02 | **-0.04** | **0.04** | - | - | - | - | - |
| | | T | 1.25 | **-2.19** | **1.97** | - | - | - | - | - |
| | | p | 0.214 | **0.029** | **0.050** | - | - | - | - | - |
| | | 95% CI | [-0.01, 0.04] | **[-0.08, -0.00]** | **[0.00, 0.08]** | - | - | - | - | - |
| | Model 2 | β | 0.01 | **-0.04** | **0.05** | -0.02 | 0.17 | **0.04** | - | - |
| | | T | 0.9 | **-2.17** | **2.22** | -0.96 | 1.46 | **2.26** | - | - |
| | | p | 0.370 | **0.031** | **0.027** | 0.337 | 0.146 | **0.024** | - | - |
| | | 95% CI | [-0.01, 0.04] | **[-0.08, -0.00]** | **[0.01, 0.09]** | [-0.05, 0.02] | [-0.06, 0.41] | **[0.00, 0.07]** | - | - |
| | Model 3 | β | 0.00 | **-0.04** | 0.04 | -0.02 | 0.24 | **0.03** | 0.00 | 0.00 |
| | | T | 0.21 | **-2.0** | 1.45 | -0.88 | 1.59 | **2.2** | 0.44 | 0.84 |
| | | p | 0.832 | **0.046** | 0.148 | 0.377 | 0.112 | **0.029** | 0.659 | 0.400 |
| | | 95% CI | [-0.03, 0.04] | **[-0.09, -0.00]** | [-0.01, 0.09] | [-0.05, 0.02] | [-0.06, 0.54] | **[0.00, 0.07]** | [-0.00, 0.00] | [-0.00, 0.00] |
| Short-term memory | Model 1 | β | 0.01 | 0.00 | 0.01 | - | - | - | - | - |
| | | T | 1.05 | 0.32 | 1.06 | - | - | - | - | - |
| | | p | 0.294 | 0.746 | 0.288 | - | - | - | - | - |
| | | 95% CI | [-0.01, 0.02] | [-0.02, 0.03] | [-0.01, 0.04] | - | - | - | - | - |
| | Model 2 | β | 0.01 | 0.00 | 0.02 | -0.02 | 0.01 | 0.01 | - | - |
| | | T | 1.05 | 0.33 | 1.16 | -1.93 | 0.17 | 1.35 | - | - |
| | | p | 0.297 | 0.744 | 0.247 | 0.054 | 0.865 | 0.179 | - | - |
| | | 95% CI | [-0.01, 0.02] | [-0.02, 0.03] | [-0.01, 0.04] | [-0.04, 0.00] | [-0.14, 0.16] | [-0.01, 0.03] | - | - |
| | Model 3 | β | 0.01 | 0.00 | 0.01 | -0.02 | 0.03 | 0.01 | 0.00 | 0.00 |
| | | T | 0.49 | 0.16 | 0.67 | -1.87 | 0.33 | 1.32 | 0.30 | 0.06 |
| | | p | 0.622 | 0.870 | 0.502 | 0.062 | 0.739 | 0.189 | 0.766 | 0.952 |
| | | 95% CI | [-0.02, 0.03] | [-0.03, 0.03] | [-0.02, 0.05] | [-0.04, 0.00] | [-0.16, 0.23] | [-0.01, 0.03] | [-0.00, 0.00] | [-0.00, 0.00] |

| | | | Plant-dominant Diet | Meat-dominant Diet | Western-Style Diet | Age | Sex | Total years of education | Energy intake | MVPA |
|---|---|---|---|---|---|---|---|---|---|---|
| Executive function | Model 1 | β | -0.01 | -0.01 | 0.01 | - | - | - | - | - |
| | | T | -0.99 | -1.48 | 1.29 | - | - | - | - | - |
| | | p | 0.324 | 0.140 | 0.198 | - | - | - | - | - |
| | | 95% CI | [-0.05, 0.05] | [-0.02, 0.01] | [-0.03, 0.00] | - | - | - | - | - |
| | Model 2 | β | -0.00 | -0.01 | 0.01 | **-0.04** | **-0.23** | 0.01 | - | - |
| | | T | -0.02 | -1.43 | 0.88 | **-5.32** | **-3.96** | 1.88 | - | - |
| | | p | 0.985 | 0.153 | 0.379 | **<0.001** | **<0.001** | 0.061 | - | - |
| | | 95% CI | [-0.01, 0.01] | [-0.03, 0.01] | [-0.01, 0.03] | **[-0.06, -0.03]** | **[-0.06, -0.03]** | [-0.34, -0.11] | | |
| | Model 3 | β | -0.01 | **-0.02** | -0.00 | **-0.04** | **-0.19** | 0.01 | 0.00 | -0.00 |
| | | T | -0.77 | **-1.96** | -0.11 | **-5.28** | **-2.51** | 1.83 | 1.29 | -0.99 |
| | | p | 0.443 | **0.050** | 0.915 | **<0.001** | **0.013** | 0.068 | 0.199 | 0.322 |
| | | 95% CI | [-0.02, 0.03] | **[-0.03, 0.03]** | [-0.02, 0.05] | **[-0.04, 0.00]** | **[-0.16, 0.23]** | [-0.01, 0.03] | [-0.00, 0.00] | [-0.00, 0.00] |
| Processing speed | Model 1 | β | 0.01 | **-0.03** | 0.03 | - | - | - | - | - |
| | | T | 1.1 | **-2.09** | 1.79 | - | - | - | - | - |
| | | p | 0.273 | **0.038** | 0.075 | - | - | - | - | - |
| | | 95% CI | [-0.01, 0.03] | **[-0.06, -0.00]** | [-0.00, 0.06] | - | - | - | - | - |
| | Model 2 | β | 0.01 | **-0.03** | 0.03 | **-0.03** | -0.10 | -0.00 | - | - |
| | | T | 1.41 | **-2.05** | 1.59 | **-2.67** | -1.08 | -0.17 | - | - |
| | | p | 0.160 | **0.042** | 0.112 | **0.008** | 0.279 | 0.863 | - | - |
| | | 95% CI | [-0.01, 0.03] | **[-0.06, -0.00]** | [-0.01, 0.06] | **[-0.06, -0.01]** | [-0.27, 0.08] | [-0.03, 0.02] | - | - |
| | Model 3 | β | 0.02 | -0.01 | **0.05** | **-0.03** | -0.13 | -0.00 | -0.00 | **0.00** |
| | | T | 1.68 | -0.72 | **2.27** | **-2.55** | -1.19 | -0.14 | -1.59 | **3.24** |
| | | p | 0.094 | 0.470 | **0.024** | **0.011** | 0.235 | 0.889 | 0.112 | **0.001** |
| | | 95% CI | [-0.00, 0.05] | [-0.04, 0.02] | **[0.01, 0.09]** | **[-0.06, -0.01]** | [-0.35, 0.09] | [-0.02, 0.02] | [-0.00, 0.00] | **[0.00, 0.00**] |

**Table 6**

Sensitivity analysis using raw data outcomes for cognitive functions with diet and MetSSS interaction terms: Model 1 (diet and interaction terms), Model 2 (Model 1 and age, sex, total years of education), and Model 3 (Model 2 and energy intake and MVPA)

| | | | Plant Diet | Meat Diet | Western Diet | MetSSS | Plant *MetSSS | Meat *MetSSS | Western *MetSSS | Age | Sex | Education | Energy intake | MVPA |
|---|---|---|---|---|---|---|---|---|---|---|---|---|---|---|
| Long-Term Memory | Model 1 | β | 0.00 | 0.02 | 0.00 | -0.03 | 0.01 | **-0.03** | 0.02 | | | | | |
| | | T | 0.03 | 0.76 | 0.03 | -1.11 | 1.08 | **-2.58** | 1.79 | | | | | |
| | | *p* | 0.935 | 0.455 | 0.868 | 0.321 | 0.288 | **0.012** | 0.082 | | | | | |
| | Model 2 | β | 0.00 | 0.02 | 0.00 | -0.02 | 0.01 | **-0.03** | 0.02 | -0.02 | 0.15 | **0.03** | | |
| | | T | -0.1 | 0.73 | 0.1 | -1.02 | 1.3 | **-2.56** | 1.89 | -1.01 | 1.3 | **2.08** | | |
| | | p | 0.923 | 0.473 | 0.861 | 0.312 | 0.319 | **0.013** | 0.066 | 0.312 | 0.193 | **0.038** | | |
| | Model 3 | β | 0.00 | 0.02 | 0.00 | -0.02 | 0.01 | **-0.03** | 0.02 | -0.02 | 0.19 | **0.03** | 0.00 | 0.00 |
| | | T | -0.2 | 0.59 | 0.08 | -0.83 | 0.9 | **-2.49** | 1.8 | -1.02 | 1.25 | **2.11** | 0.17 | 0.46 |
| | | *p* | 0.838 | 0.559 | 0.867 | 0.461 | 0.379 | **0.015** | 0.082 | 0.309 | 0.214 | **0.036** | 0.864 | 0.651 |
| Short-term memory | Model 1 | β | -0.01 | 0.01 | 0.02 | **-0.04** | 0.01 | 0.00 | 0.00 | | | | | |
| | | T | -0.44 | 0.73 | 0.69 | **-1.89** | 1.3 | -0.65 | 0.28 | | | | | |
| | | p | 0.523 | 0.446 | 0.500 | **0.044** | 0.121 | 0.499 | 0.764 | | | | | |
| | Model 2 | β | -0.01 | 0.02 | 0.01 | -0.03 | 0.01 | -0.01 | 0.00 | -0.02 | 0.02 | 0.01 | | |
| | | T | -0.45 | 0.78 | 0.61 | -1.65 | 1.33 | -0.76 | 0.43 | -1.65 | 0.29 | 1.14 | | |
| | | *p* | 0.655 | 0.434 | 0.541 | 0.101 | 0.191 | 0.448 | 0.669 | 0.101 | 0.769 | 0.255 | | |
| | Model 3 | β | 0.00 | 0.02 | 0.01 | -0.03 | 0.01 | 0.00 | 0.00 | -0.02 | 0.00 | 0.01 | 0.00 | 0.00 |
| | | T | -0.25 | 0.81 | 0.39 | -1.7 | 1.11 | -0.68 | 0.48 | -1.86 | 0.04 | 1.24 | 0.04 | -0.31 |
| | | *p* | 0.807 | 0.422 | 0.695 | 0.094 | 0.274 | 0.499 | 0.633 | 0.064 | 0.968 | 0.215 | 0.965 | 0.754 |

| | | | Plant Diet | Meat Diet | Western Diet | MetSSS | Plant* MetSSS | Meat* MetSSS | WSD* MetSSS | Age | Sex | Education | Energy intake | MVPA |
|---|---|---|---|---|---|---|---|---|---|---|---|---|---|---|
| Executive Function | Model 1 | β | **-0.02** | -0.02 | 0.00 | -0.01 | **0.01** | 0.00 | 0.00 | | | | | |
| | | T | **-2.49** | -1.02 | 0.19 | -0.8 | **2.26** | 0.01 | 0.8 | | | | | |
| | | p | **0.019** | 0.309 | 0.851 | 0.429 | **0.033** | 0.921 | 0.43 | | | | | |
| | Model 2 | β | **-0.02** | -0.01 | -0.01 | **-0.04** | **0.01** | -0.00 | 0.01 | **-0.04** | **-0.23** | 0.01 | | |
| | | T | **-2.32** | -0.88 | -0.5 | **-5.18** | **2.9** | -0.31 | 1.35 | **5.18** | **-3.99** | 1.9 | | |
| | | p | **0.029** | 0.382 | 0.618 | **<0.001** | **0.007** | 0.759 | 0.181 | **<0.001** | **<0.001** | 0.058 | | |
| | Model 3 | β | **-0.03** | -0.02 | -0.02 | -0.01 | **0.01** | -0.00 | 0.01 | **-0.05** | **-0.21** | 0.01 | 0.00 | -0.00 |
| | | T | **-2.4** | -1.33 | -0.97 | -0.7 | **2.78** | -0.01 | 1.38 | **-5.44** | **-2.84** | 1.84 | 1.18 | -1.31 |
| | | p | **0.021** | 0.186 | 0.336 | 0.462 | **0.009** | 0.880 | 0.172 | **<0.001** | **0.005** | 0.066 | 0.238 | 0.199 |
| Processing speed | Model 1 | β | **-0.02** | -0.02 | 0.00 | -0.01 | **0.01** | 0.00 | 0.00 | | | | | |
| | | T | **-2.49** | -1.02 | 0.19 | -0.8 | **2.26** | 0.01 | 0.8 | | | | | |
| | | p | **0.019** | 0.309 | 0.851 | 0.429 | **0.033** | 0.921 | 0.430 | | | | | |
| | Model 2 | β | -0.00 | -0.02 | 0.01 | **-0.03** | 0.01 | -0.00 | 0.00 | **-0.03** | -0.12 | -0.00 | | |
| | | T | -0.16 | -1 | 0.56 | **-2.6** | 1.36 | -0.05 | 0.48 | **2.6** | -1.34 | -0.13 | | |
| | | p | 0.770 | 0.319 | 0.576 | **0.010** | 0.198 | 0.951 | 0.635 | **0.010** | 0.181 | 0.895 | | |
| | Model 3 | β | 0.01 | -0.01 | 0.05 | -0.02 | 0.01 | 0.00 | 0.00 | **-0.03** | -0.16 | -0.00 | -0.00 | **0.00** |
| | | T | 0.49 | -0.34 | 1.7 | -1.02 | 1.42 | 0.01 | 0.04 | **-2.56** | -1.45 | -0.22 | -1.77 | **2.9** |
| | | p | 0.633 | 0.736 | 0.092 | 0.377 | 0.176 | 0.962 | 0.874 | **0.011** | 0.149 | 0.824 | 0.079 | **0.004** |

**Sensitivity analysis with components created with oblimin rotation**

Substituting the dietary scores with principal components rotated using oblimin (compared to varimax) did not significantly alter the patterns observed in the associations between dietary patterns and cognitive outcomes. The results remained largely consistent across different models, suggesting robustness in the observed relationships irrespective of the rotation method.

The meat-dominant diet consistently showed negative associations with long-term memory across all models. The Western-style diet did not show any significant associations with long-term memory. or processing speed when using the PCA with oblimin rotation. However, there was a novel association, where a meat-dominant diet was associated with slower processing speed (Model 1: β = -0.037, 95% CI [-0.069, -0.005], $p$ = .022) and this persisted in Model 2 (β = -0.036, 95% CI [-0.068, -0.004], $p$ = .026) but not model 3.

In the oblimin rotation sensitivity analysis, the interaction between the meat-dominant diet and MetSSS showed a significant association with decreased long-term memory performance in Model 1 and Model 2, but not in Model 3. Similarly, the interaction between the plant-dominant diet and MetSSS significantly influenced executive function scores in Model 2, but not in Model 3.
